# Exosomes in Angiogenesis and Anti-angiogenic Therapy in Cancers

**DOI:** 10.3390/ijms21165840

**Published:** 2020-08-14

**Authors:** Wioletta Olejarz, Grażyna Kubiak-Tomaszewska, Alicja Chrzanowska, Tomasz Lorenc

**Affiliations:** 1 Department of Biochemistry and Pharmacogenomics, Faculty of Pharmacy, Medical University of Warsaw, 02-097 Warsaw, Poland; wioletta.olejarz@wum.edu.pl (W.O.); grazyna.kubiak-tomaszewska@wum.edu.pl (G.K.-T.); 2 Centre for Preclinical Research, Medical University of Warsaw, 02-097 Warsaw, Poland; 3 Chair and Department of Biochemistry, Medical University of Warsaw, ul. Banacha 1, 02-097 Warsaw, Poland; alicja.chrzanowska@wum.edu.pl; 4 1st Department of Clinical Radiology, Medical University of Warsaw, ul. Chałubińskiego 5, 02-004 Warsaw, Poland

**Keywords:** angiogenesis, exosomes, anti-angiogenic therapy, extracellular vesicles

## Abstract

Angiogenesis is the process through which new blood vessels are formed from pre-existing ones. Exosomes are involved in angiogenesis in cancer progression by transporting numerous pro-angiogenic biomolecules like vascular endothelial growth factor (VEGF), matrix metalloproteinases (MMPs), and microRNAs. Exosomes promote angiogenesis by suppressing expression of factor-inhibiting hypoxia-inducible factor 1 (HIF-1). Uptake of tumor-derived exosomes (TEX) by normal endothelial cells activates angiogenic signaling pathways in endothelial cells and stimulates new vessel formation. TEX-driven cross-talk of mesenchymal stem cells (MSCs) with immune cells blocks their anti-tumor activity. Effective inhibition of tumor angiogenesis may arrest tumor progression. Bevacizumab, a VEGF-specific antibody, was the first antiangiogenic agent to enter the clinic. The most important clinical problem associated with cancer therapy using VEGF- or VEFGR-targeting agents is drug resistance. Combined strategies based on angiogenesis inhibitors and immunotherapy effectively enhances therapies in various cancers, but effective treatment requires further research.

## 1. Introduction

Angiogenesis and inflammation are processes that play important roles in the development of cancer, from the initiation of carcinogenesis, the carcinoma in situ stage, to the advanced stages of cancer [1]. Excessive abnormal angiogenesis has a central role in tumor progression. It is induced by an imbalance between pro- and anti-angiogenic factors, dominated by tissue hypoxia-triggered overproduction of vascular endothelial growth factor (VEGF) [2]. Proliferation and metastatic spread of cancer cells depends on an adequate supply of oxygen and nutrients and the removal of waste products [3]. Tumor cells utilize different strategies to communicate with neighboring tissues for facilitating tumor progression; one of these strategies is a release of exosomes [4,5]. Tumor cells, as well as immune cells, secrete exosomes that affect the activation of immune cells and immune surveillance [6]. Exosomes, harboring various cargoes that can accelerate angiogenesis, play an important role in cancer invasiveness [7]. According to numerous studies, by releasing exosomes, tumor cells are able to promote tumor epithelial–mesenchymal transition, angiogenesis, and immune escape [8]. Exosomes can shuttle diverse biomolecules like microRNAs, DNA fragments, proteins, lipids, and even pharmacological compounds from a donor cell to recipient cells [9]. There is evidence that noncoding RNAs, especially long non-coding RNAs (lncRNAs) and microRNAs, play important roles in the regulation of angiogenesis [10]. It has been shown that tumor vasculogenesis and progression after anti-angiogenic therapies (AATs) and anti-autophagic therapies are due to cross-talk between endothelial and tumor cells via VEGF-enriched exosomes [11]. Manipulation of angiogenesis has become an attractive approach for cancer therapy since the introduction of the first angiogenesis inhibitor, bevacizumab, for metastatic colorectal cancer therapy [12]. The implementation of anti-angiogenic strategies faces several pitfalls due to the potential involvement of multiple pro-angiogenic factors and modulatory effects of the innate and adaptive immune system [2]. This review will consider exosomes in the context of tumor angiogenesis and the limited efficacy of current anti-angiogenic approaches.

## 2. Characteristics of Exosomes

**Figure 1:**
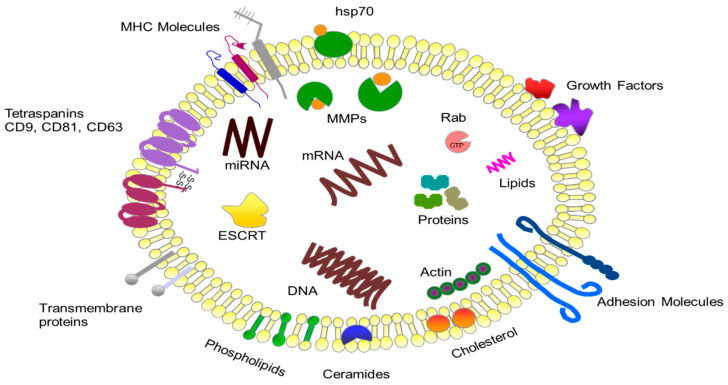
The structure and content of exosomes.

Exosomes belong to the large group of small extracellular vesicles, including oncosomes, microvesicles, exosomes and apoptotic bodies [13]. They vary in size considerably with a range of 30–100 nm and density of 1.13–1.19 g/mL [14,15]. Exosomes are carriers of numerous proteins, lipids and nucleic acids (Figure 1). The content of exosomes depends on the composition of the cells from which they originate [16,17,18]. Exosomes are secreted by both normal and pathological cells. The process of creating exosomes involves many stages. These include the gradual formation of interstitial vesicles (ILVs) within the intracellular multivascular body (MVB) and their secretion into the intercellular space as small exosomes [19]. This process can take place via three paths: with the participation of the endosomal sorting complex required for transport function (ESCRT), as a result of ceramide-dependent lateral phase separation and coalescence of tetraspanin-rich membrane microdomains (TEM) present in a raft, or with the participation of ALG-2 interacting protein X (ALIX) and lysobisphosphatidic acid (LBPA) [15,20,21]. These processes involve a significant amount of formed ILVs in exogenous biogenesis (ILVs are derived from cholesterol-rich MVBs). In addition, the vesicles participate in the biogenesis of lysosome-associated organelles in specialized cells and the transport of specific proteins to membranes, and are subject to degradation within lysosomes (IVLs derived from cholesterol-poor MVBs) [22,23]. Exosomes have been found in whole blood, plasma, cerebrospinal fluid, urine, semen, saliva, synovial fluid, milk, tears, nasal secretions and feces [24,25,26,27].

The first reports of exosomes classified them as structures that enable the removal of unnecessary metabolites from cells. Currently, they are considered important elements of intercellular communication pathways, where they modulate the tissue’s microenvironment [16,28]. Exosomes are involved in physiological processes like tissue repair and regeneration or development of the nervous system, as well as pathological processes (cancer angiogenesis and progression, immune system regulation in infection, atherosclerosis, and neurodegenerative diseases, such as Parkinson’s, Alzheimer’s and amyotrophic lateral sclerosis) [22,23,29,30,31].

## 3. Mechanism of Angiogenesis

Cancer cells have a special mechanism for blood vessel development, which involves either incorporation of host blood vessels into the tumor or a unique ability to express the endothelial cell (EC) phenotype and form structures similar to vessels. Pathologic angiogenesis, according to Folkman’s postulate [32], is a necessary step for a cancerous tumor of a volume more than 1 mm^3^ to develop. At the initial stage of progression, tumor nodules are clusters of about 1 million cells. At this stage, a balance is found between the process of proliferation and apoptosis (cell death), and this period may take months or even years [33]. During this phase, the development of tumor tissue is independent of the vascular network structure and oxygen, as well as nutrients and growth factors. They reach tumor cells mainly as a result of diffusion from nearby vessels. At a later stage of development, necrosis occurs in the central zones of the tumor and without further blood supply it would be impossible to grow [34]. Therefore, the next stage of carcinogenesis is associated with the obtainment of an angiogenic phenotype by cancer cells—during the so-called angiogenic transition, i.e., a state of permanent, genetic modifications, leading to uncontrolled production of proangiogenic factors [3]. Newly formed vessels are different from normal vessels, they have the wrong shape and size, and are irregular, immature, tortuous and distended [35,36]. They also have numerous tabs that enter the lumen of the vessels. They show incomplete arteriovenous differentiation and incomplete differentiation of perivascular space. The blood flow in such vessels is slow. The connections between the cells are wide, and numerous pores appear, which is why they are also more permeable. This increases the interstitial pressure in the tumor [37,38]. In addition, it has been observed that the vessels inside the tumor differ from those outside, because the latter are more like normal vessels. Vessels in tumors are subject to constant reorganization—they arise and recede. Their course and location in tumors change over time. This causes extensive transformations within the tumor and changes in the localization of the necrotic area. There has been a lot of data indicating that cancer vessels do not fulfill their basic physiological functions and do not supply cancer cells with sufficient oxygen [39]. Tumor neovascularization provides the opportunity for tumor growth by supplying cancer with oxygen, nutrients, and metabolite replacement, but also because endothelial cells of blood vessels contribute to the increase in tumor volume. Adequate blood supply to the tumor promotes the entry of tumor cells into the bloodstream and, consequently, initiating the metastasis process. Therefore, it can be stated that determining the number of capillary blood vessels in histopathological preparations of tumor material has considerable diagnostic and prognostic significance, as it allows one to determine the risk of metastasis [40].

There are two main processes involved in the formation of blood vessels, namely vasculogenesis and angiogenesis. Vasculogenesis differs from angiogenesis in one aspect: angiogenesis is the formation of new blood vessels from pre-existing ones, while vasculogenesis is the formation of new blood vessels from endothelial cell precursors or angioblasts from the mesoderm and further formation of elementary capillary plexuses that will finally develop into mature vessels [41]. 

**Figure 2:**
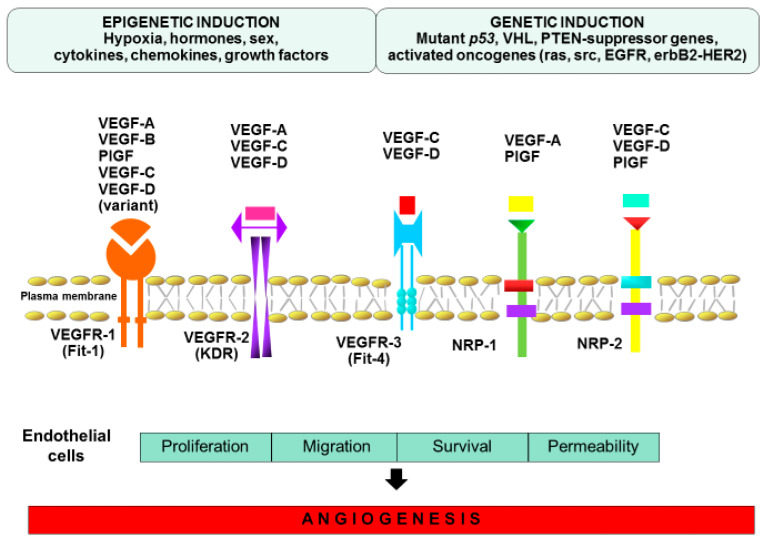
Epigenetic and genetic induction of angiogenesis.

The activity of the angiogenesis process is the result of the action of pro- and anti-angiogenic factors, which also act as markers of this process. Under haemostasis, the balance of these factors tilts slightly towards angiogenesis [42]. Physiological neovascularization is a slow process in adults. In the resting vascular system, only about 0.5% of endothelial cells show mitotic activity, and the duration of the cycle of angiogenic activity of the endothelium is 1000 days [32]. Angiogenesis requires the transition of endothelial cells from the resting to the activated state. There are two basic mechanisms of angiogenesis: the first one is sprouting angiogenesis, which is the most common and can be described as germination of endothelial cells, and the second one is intussusceptive angiogenesis–invagination into the vessel’s lumen [43]. The literature suggests that sprouting angiogenesis is caused by hypoxia, whereas intussusceptive angiogenesis relies on hemodynamic factors [44]. The sprouting process is regulated by the balance between proangiogenic agents, including VEGF, and quiescence-inducing factors, such as pericyte contact and VEGF inhibitors [45]. At the onset, angiogenic expansion of primary capillary plexuses occurs, which leads to capillary vessel development and then capillary vessel system formation, together with physiological expansion of surrounding tissues [46]. This series of molecular and physiologic changes will lead to variability in the epithelium. Next, further maturation of vessel structures takes place, meaning their diameter and wall thickness will increase in a process called arteriogenesis. Primarily it is wall cells that multiply and acquire more specialized features such as contractibility [47]. The mechanism of angiogenesis is a multistage process and many growth factors, substances and types of cells take part in it (Figure 2). When cells in a dormant vessel sense angiogenetic signals the surrounding pericytes part from the vessel’s wall and epithelial cells relax connections between each other, transforming into “tip cells” that are localized in the growing end of the vessel that has long filopodia [48]. Because of these filopodia, the vessel cell recognizes the concentration gradient of proangiogenic agents released by other cells. Proliferation of stalk cells localized in the growing end of the vessel is controlled inter alia by NOTCH, WNT, placenta growth factor (PlGF), and fibroblast growth factor (FGF) [49,50]. Specific apical cells also excrete inhibiting signals into their surroundings and in this way halt uncontrolled migration towards angiogenic signals. After apical cells, there are endothelial stalk cells with fewer filopodia, stretching, proliferating, and creating the vessel lumen through tight and adhering connections with neighboring endothelial cells to aid the process of sprouting [51]. To achieve a functional and mature blood vessel, migration, as well as proliferation of endothelial cells, needs to be stopped. Finally, the recruitment of pericytes and endothelial smooth muscles must take place. Pericytes will build capillary vessel walls and stabilize newly developed vessels [52]. Various signaling pathways are involved in the endothelium/pericyte cross-talk, which promote endothelial retirement and new vessel stabilization. The best known are angiopoietin-1 (ANG-1)/Tie2, transforming growth factor (TGF-β)/TGF-R, and ephrinB2/EphB4 [53,54,55]. However, intussusceptive angiogenesis seems to be characterized by low proliferative potential and poor extracellular matrix degradation. Hemodynamic factors appear to contribute to intussusceptive angiogenesis and additionally VEGF-A seems to play an important role in shear stress-based splitting of capillaries [56]. This process includes the splitting of vessels through the inclusion of tissue pillars. Afterwards, these pillars are surrounded by supporting cells, such as fibroblasts and pericytes, which create extracellular matrix. Accordingly, this event leads to part of the vessel splitting, creating two new ones [57].

Moreover, vessel formation in tumors can occur by recruitment of circulating endothelial progenitor cells or bone marrow-derived hematopoietic cells, which may differentiate and accumulate into clusters called blood islands [58]. The angioblasts located at the periphery of the blood islands, are precursors for endothelial cells, while those at the center differentiate to hematopoietic cells [59]. Vasculogenesis is initiated by interaction between VEGF and the tumor microenvironment, which may mobilize VEGFR-2+ EPCs in the bone marrow. To mobilize endothelial progenitor cells (EPCs) and promote neovascularization, tumors also secrete adiponectin, chemokines C–C motif ligand (CCL)2 and CCL5, and the hypoxia responsive chemokine SDF-1 [60,61,62].

Through the vascular mimicry process, aggressively growing tumor cells can form structures similar to vessels without participation of endothelial cells [63]. To form and stabilize vessels, the endothelial-like tumor cells can secrete heparan sulfate, collagens IV and VI, proteoglycans, tissue transglutaminase antigen 2 and laminin [64]. Increased vascular mimicry has been observed following anti angiogenic therapy and is considered a marker for poor prognosis of cancer progression [65,66]. It was observed that tumor endothelial cells can contain similar somatic mutations to malignant tumor cells [67]. This process contains trans-differentiation of cancer stem cells to endothelial cells and vascular smooth muscle-like cells [68]. It was found that human glioma stem cells developed vessels with endothelial cells expressing proteins such as VEGFR-2, CD34 and CD144 [69].

## 4. Endogenous Regulators of Angiogenesis

Regulators of angiogenesis are a very large and heterogenous group with a wide range, including polypeptides, metabolites and hormones that collaborate in order to form new blood vessels [70]. A major factor of angiogenesis under normal conditions and in a disease state is vascular endothelial growth factor A (VEGF-A) [71]. It is a member of the gene factors family, which includes VEGF-B, VEGF-C, VEGF-D, VEGF-E and placenta growth factor (PlGF). These factors show different affinities to tyrosine kinase receptors (VEGFR) -1, -2 and -3 [72]. VEGF-A may bind to VEGFR-2 (mainly occurring on blood vessel ECs) and contributing to the process of angiogenesis, whereas VEGF-C and -D preferentially bind to VEGFR-3, which is predominantly found on lymphatic ECs, resulting in proliferation of lymphatic vessels [71]. VEGF promotes the cancer stem cells’ functionality and may initiate tumorigenesis by activation of epithelial–mesenchymal transition (EMT) [73]. This leads to a loss of cell polarity and cytoskeletal changes, which cause an increase in cell motility and a decrease in cell adhesion through loss of E-cadherin and ZO-1 [74]. To allow endothelial cell migration and formation of capillary sprouts, basal membrane degradation must occur. For this process, matrix metalloproteinases, MMP-2, MMP-9, and urokinase plasminogen activator (uPA) are responsible [75]. Expression of membrane-type matrix metalloproteinases (MT–MMPs) promotes VEGF-mediated cell invasion. VEGF also induces vascular permeability, which may facilitate the escape of tumor cells into the bloodstream and promote distant metastases [76]. An interesting fact is that the role of PlGF in modulating angiogenesis process is not so obvious. PlGF may initiate cross-talk between VEGFR-1 and VEGFR-2 [77], while other studies reported its antiangiogenic properties [78] (Figure 2). Proteolysis mediated by plasmin (PLA) is an essential feature of angiogenesis and cell invasion [79]. Whereas antithrombin is a key inhibitor of the coagulation cascade, it may also have an anti-angiogenic function [80].

Other angiogenesis inductors are platelet-derived growth factors (PDGF)-B and -C and fibroblast growth factor (FGF)-1 and -2, which may bind to their respective receptors on blood vessel ECs and induce their proliferation and migration. The PDGF family includes four heparin-binding polypeptide growth factors (A, B, C, and D). PDGF binds and transduces signals through two cell-surface tyrosine kinase receptors, PDGFRα and PDGFRβ [81]. It can lead to a promotion of vessel maturation, recruitment of pericytes and VEGF upregulation. Among all members of the PDGF family, the most noteworthy for its potent angiogenic activity in vivo is the PDGF-B/PDGFRβ axis [82,83]. The fibroblast growth factor (FGF) family has 22 members and most of them show high affinity to tyrosine kinase receptors FGFR-1, FGFR-2, FGFR-3 and FGFR-4. FGF expression in tumors is responsible for resistance to anti-angiogenic therapy [84,85]. Both FGF-2, also known as basic FGF (bFGF), and VEGF can initiate angiogenesis by increasing secretion of MMPs, plasminogen activator and collagenase, responsible for the degradation and rebuilding of extracellular matrix [86]. A recent study reported that FGF modulates endothelial metabolism driven by MYC-dependent glycolysis signaling, important for blood and lymphatic vascular development [87].

**Figure 3:**
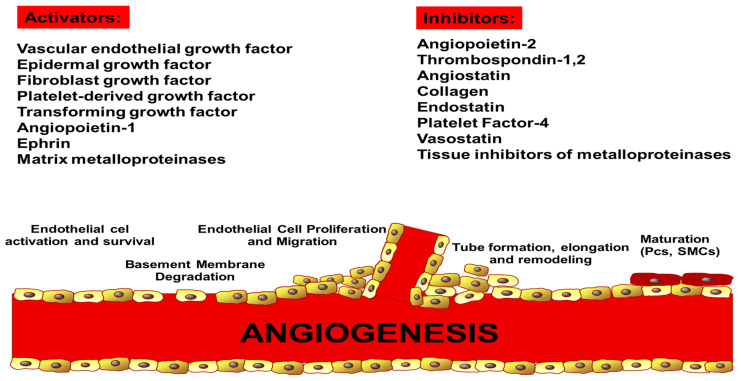
Endogenous regulators of angiogenesis.

Another important angiogenic factor is angiopoietin. The angiopoietin family comprises ligands ANGPT-1, ANGPT-2, and ANGPT-4. Their signaling is mediated by endothelial receptor tyrosine kinases, TIE-1 and the more well known TIE-2. Interestingly, both ligands (ANGPT-1 and ANGPT-2) bind to TIE-2, but have various effects [88,89]. ANGPT-1 initiates vessel maturation and newly formed vessel stability by the Akt/survivin pathway [90]. On the contrary, ANGPT-2 may induce vessel destabilization, pericyte separation, vessel germination and angiogenesis [88]. Increased ANGPT-2 expression has been reported in tumor-associated vessels of a few human cancers in response to hypoxia and VEGF action [91]. The angiopoietin (ANGPT)–TIE system is crucial for the angiogenic switch in tumors, and together with VEGF-A promotes the initiation of angiogenesis and maturation of new vessels [92]. On the other hand, there is a wide range of antiangiogenetic factors such as thrombospondin-1 (TSP1), which is a large glycoprotein present in ECM, or proteolytic product of collagen XVIII, called endostatin [43,44] (Figure 3).

Other angiogenesis inhibitors are interferon-alpha and -beta and angiostatin, a cleavage product of plasmin [45,93]. A balance between angiogenesis promoters and inhibitors is regulated by different pathways. Hypoxia, cellular nutrient deficiency, hypoglycaemia, and metabolic acidosis, among others, are cell environmental factors contributing to a proangiogenic imbalance, which frequently takes place at the gene level due to oncogene activation or tumor suppressor gene inactivation [7,94]. Tumor cells trigger this imbalance, while inflammatory cells infiltrate the surrounding tissues. This can lead to angiogenic switches and progression from hyperplasia to hypervascularised tumor. The study of Rip1Tag2 mice showed the phases of carcinogenesis, from normal cells to hyperplasia, and adenoma to highly advanced carcinoma. VEGF-A was shown to be the main regulator of EC proliferation, migration, and vessel formation [95]. Angiogenesis occurred preferentially in mice that overexpressed human VEGF-A165 in pancreatic β-cells, even at an early stage of carcinogenesis [96]. In contrast, inhibiting VEGF-A caused suppression of the angiogenic switch and tumor growth [97]. 

Creation of new blood vessels, which begins at an early stage of cancer development, is associated with the number of exosomes produced by cancer. Proangiogenic effects were observed with use of exosomes originating from cancer cell lines, as well as exosomes isolated from cancer patients blood samples and urine collected from urinary bladder cancer patients [98]. It is interesting that gliomas, for example, have richer vasculature compared to other solid tumors. Both in vitro and in vivo studies showed that EVs excreted by gliomas contain angiogenic proteins [99,100]. Moreover, other solid tumors, such as pancreatic cancer and breast cancer, create exosomes that induce neovascularization [7]. Aside from solid tumors, it was also shown that exosomes are produced by chronic myelogenous leukemia cells and that they have an impact on blood vessel creation through a direct interaction with ECs [101].

## 5. Exosome Uptake by ECs 

Exosomes can interact with target cells such as ECs, but also with immune cells to initiate and facilitate angiogenesis. Uptake of tumor-derived exosomes by normal endothelial cells activates angiogenic signaling pathways in endothelial cells and stimulates new vessel formation [102]. Exosomes can affect T cells through direct receptor–ligand interactions, but in ECs, exosomes usually use the internalization pathway [103]. ECs internalize exosomes produced by cancer cells within 2–4 h. This was confirmed, among others, by studies where ECs easily captured PKH26-dyed exosomes during the first 4 h [104]. Immediately after internalization, exosomes are directed to the perinuclear zone. When tubules are formed in vitro, exosomes move to the periphery of the cell and enter advanced pseudopods. After complete remodeling, adjacent ECs probably transport exosomes to other ECs and to other cells in the TME (tumor microenviroment) through nanoparticle structures [105].

## 6. History of Anti-Angiogenic Therapy

Since pre-clinical studies showed that tumors induce the sprouting of new vessels from the surrounding vasculature, there was great optimism that inhibition of pro-angiogenic growth factors would represent an effective antiangiogenic therapy for most tumor types [106]. Further studies in animal models established that the vascular supply can be suppressed by inhibition of VEGF [107]. Based on successful randomized trials, anti-VEGF therapeutics have entered clinical practice for the therapy of cancer. Bevacizumab, a VEGF-specific antibody, was the first antiangiogenic agent to enter the clinic, and is currently approved for use in colorectal and lung cancer treatment [108]. So far, several VEGF blockers have been approved for clinical use in cancer [109]. In addition, several multi-targeted tyrosine kinase inhibitors (TKIs), which block the signaling of pathways such as VEGF, have been approved, including sorafenib, sunitinib, pazopanib, and vandetanib [110]. Among the currently available anti-angiogenic drugs, bevacizumab, sunitinib, pazopanib, endostar, regorafenib, axitinib, sorafenib, ranibizumab, and aflibercept are the most used in the treatment of various cancer types [38].

## 7. Classification of Angiogenesis Inhibitors 

Angiogenesis inhibitors are classified into direct and indirect agents. Direct endogenous inhibitors (endostatin, arrestin, and tumstatin) target vascular ECs, but unfortunately, phase II or III clinical trials did not result in significant effects on patients [109,110]. Indirect angiogenesis inhibitors (AIs) target tumor cells or tumor-associated stromal cells and prevent the expression of pro-angiogenic factors or block their activity [109]. To develop anti-angiogenic agents, four main strategies are applied: the inhibition of endogenous factors promoting blood vessel formation, the identification and application of natural angiogenesis inhibitors, molecule inhibition promoting the invasion of surrounding tissue through tumor blood vessels, and the incapacitation of actively proliferating endothelial cells [38]. As a result, the last decade has given rise to many anti-angiogenic agents developed for cancer treatment, with at least eighty drugs being investigated in preclinical studies and phase I–III clinical trials. Despite promising preclinical results, anti-angiogenic monotherapies offer mild clinical benefits. The most important clinical problem associated with cancer therapy using VEGF- or VEFGR-targeting agents is drug resistance, as a result of clonal expansion or sub clonal evolution of tumors with the upregulation of other angiogenic factors [111]. VEGF-dependent alterations, non-VEGF pathways and stromal cell interactions are mechanisms of resistance [112]. Because recent literature highlights the variability of patient and tumor responses to anti-angiogenic drugs, predictive in vitro models that can recapitulate the drug response have been used for a personalized medicine approach [113]. In advanced cancers, the tumor develops escape strategies and quickly overcomes the inhibition of angiogenic pathways. Because of these limitations, it is crucial to identify biomarkers that are able to predict responses and prognoses related to anti-angiogenic treatment. Over recent years, extracellular vesicle involvement in tumor progression and resistance has been thoroughly considered [114]. In the next section, we will focus on the interaction between exosomes and anti-angiogenic resistance in cancer cells.

## 8. Anti-Angiogenic Therapy in the Most Common Cancers

### 8.1. Prostate Cancer

Angiogenesis in prostate cancer was initially associated with promising findings in early studies, but phase III clinical trials, mainly conducted after 2010, have offered disappointing results thus far [115]. Most anti-angiogenic clinical studies in prostate cancer have targeted VEGF-A because it was found to be overexpressed in prostate cancer and associated with poor prognosis and metastasis [115]. Some randomized phase II trials on bevacizumab, which involved patients with hormone-sensitive prostate cancer, showed improved relapse-free survival when bevacizumab was used alongside hormone-deprivation therapy [116]. In phase III, when bevacizumab was used together with docetaxel chemotherapy and prednisone hormonal therapy, some improvement in progression-free survival was observed, yet it caused no significant changes in the overall survival of metastatic, castration-resistant, prostate cancer patients [117]. Furthermore, bevacizumab is associated with increased toxicity and a greater incidence of treatment-related deaths [117]. This suggests that in hormone-resistant refractory tumors, in which conventional treatment options are particularly prone to failure, adding bevacizumab treatment does not have any clinical benefit. Even so, bevacizumab has some positive effects, especially on hormone-sensitive recurrent prostate cancer [117]. To summarize, these findings suggest that anti-angiogenic therapy has no clinical benefit when added to chemotherapy or hormonal therapy in refractory, castration-resistant prostate cancer [115]. Further possible treatment options, including direct targeting of VEGFR-2-expression, indirect inhibition of angiogenesis, and targeting the interplay between tumor or stromal cells and angiogenesis, have been evaluated. Lu et al. suggested that anti-VEGFR-2-AF is a prospective therapeutic Ab for prostate cancer treatment that inhibits angiogenesis, through vascular endothelial cells, and tumorigenesis at the same time by VEGFR-2-expressing tumor cells [118]. The therapeutic efficacy of anti-VEGFR-2-AF is currently under study in preclinical trials using solid and liquid xenograft mouse models [118]. 

### 8.2. Hepatocellular Carcinoma 

Advanced hepatocellular carcinoma (HCC) has limited treatment options, where overall survival can be improved but no cure has been found [119]. Given the highly vascular nature of HCC, anti-angiogenic therapy is currently the recommended therapy for advanced stage disease [120]. Sorafenib and lenvatinib are used as first-line treatments for advanced unresectable HCC [119]; however, both have numerous side effects [119]. Regorafenib and cabozantinib are tyrosine kinase inhibitors. They are the only anti-angiogenic drugs seen to be advantageous as a second-line therapy in patients progressing on sorafenib. Regorafenib and cabozantinib show statistically significant improvement compared to placebo in the overall survival and progression-free survival of patients [121,122].

### 8.3. Melanoma

Melanoma is amongst the cancer types where anti-angiogenic therapy has been disappointing in terms of showing no overall survival benefit in phase 3 trials [123]. Nevertheless, pre-clinical and clinical trials are being conducted to examine the effects of various anti-angiogenic experimental therapies [124]. Although most studies focus on VEGF signaling inhibition, others are aimed at determining the effect of multikinase inhibitors or the inhibition of angiogenic integrin activity [125].

### 8.4. Ovarian Cancer

The anti-angiogenic agent bevacizumab, given concomitant to combination chemotherapy followed by maintenance therapy, is considered the standard of care in patients with advanced ovarian cancer. It is given as first-line therapy and in those with platinum-sensitive recurrent ovarian cancer [126].

### 8.5. Colorectal Cancer

The second-line regimen choice in metastatic colorectal cancer is greatly dependent on the systemic therapies given as first-line treatment. Anti-angiogenic agents (e.g., bevacizumab, ramucirumab and aflibercept) are indicated for most patients. Epidermal growth factor receptor (EGFR) inhibitors do not improve survival in a second-line setting [127]. Recently, a number of new orally available multiple receptor tyrosine kinase inhibitors have been tested in late-stage clinical trials, with modest efficacy [128].

### 8.6. Breast Cancer

Although the scientific rationale for anti-angiogenics appears to be well supported, so far studies have not demonstrated clinically significant benefits of adding these therapeutic agents in breast cancer [129]. Studies conducted with anti-angiogenic agents have not yet displayed clinically significant benefits as monotherapy, in combination with chemotherapy, endocrine treatment, or maintenance therapy, whether it be in the metastatic or early setting [129]. Although small improvements in complete pathologic response and progression-free survival have been shown with bevacizumab, this did not translate into improved long-term outcomes, such as disease-free survival and overall survival [129].

### 8.7. Lung Cancer

The treatment of advanced non-small cell lung cancer includes chemoimmunotherapy or targeted therapy with TKIs. When the median overall survival was thought to be less than one year, the addition of anti-angiogenics to chemotherapy resulted in modest increases in survival. More recently, the use of anti-angiogenics has fallen out of favor with the advent of check-point inhibitors, which have shown durable long-term responses that have never been previously observed [130].

### 8.8. Pancreatic Cancer

For pancreatic cancer treatments, multiple clinical trials of anti-angiogenic agents have been carried out, yet the results are overwhelmingly disappointing [131]. Pre-clinical studies suggesting that VEGF is a therapeutic target in pancreatic cancer have offered promising results in pre-clinical studies. However, phase III trials of gemcitabine plus anti-angiogenic therapy with bevacizumab and oraxitinib (a VEGFR inhibitor) failed to reach the primary endpoint of overall survival [132]. Although improved progression-free survival was observed in a few clinical trials, to date none have shown significant prolongation of overall survival for pancreatic cancer patients [131].

### 8.9. Glioblastoma

Since the approval of bevacizumab, it has been used as a second-line therapy in glioblastoma multiforme [133]. Bevacizumab may be beneficial in prolonging progression-free survival, but the routine addition of bevacizumab to standard therapy for newly diagnosed glioblastoma is not recommended in clinical practice [134].

The results of key phase III trials for anti-angiogenic therapy efficacy in patients with cancers are summarized in Table 1.

## 9. Tumor-Derived Exosomes in Angiogenesis

One of the factors determining the development of cancer and its progression is a sufficient supply of oxygen and nutrients [151]. At the initial stage of tumor development, the blood vessels in the tumor microenvironment are quite poorly organized. Vascular homeostasis disorder in conditions of dominance of proangiogenic signaling activates angiogenously inactive clusters of cancer cells. This mechanism is referred to as the “angiogenesis switch”. In the TEM, this condition is achieved by transferring angiogenic factors from cancer cells to endothelial cells [152]. As a result, the tumor is vascularized through: (1) the formation of new blood vessels in the tumor structure using circulating progenitor cells, bone marrow-derived hematopoietic cells (vasculogenesis) and cancer cells (vasculogenic mimicry and transdifferentation of cancer cells); or (2) co-optation of existing blood vessels (sprouting angiogenesis and intussusceptive angiogenesis) [42]. In turn, intensification of angiogenesis stimulates tumor growth and metastasis [70,153].

Exosomes, as transporters of numerous biomolecules, mediating communication between different types of cells, play an important role in the process of angiogenesis. According to ExoCarta data, 1116 types of lipids, 9796 subsets of proteins and 6246 types of mRNA in exosomes have been found so far. Exosomes, by providing numerous pro- and anti-angiogenic factors such as mRNA, miRNA and proteins, reprogram recipient cells by introducing changes in their functional profile [154]. One of the first reports on the role of TEX in angiogenesis concerned research on glioblastoma and colon cancer. Exosomes have been shown to be intensively absorbed by vessel cells, thereby promoting angiogenesis [154,155]. The question is what factors present in exosomes determine tumor angiogenesis?

**Figure 4:**
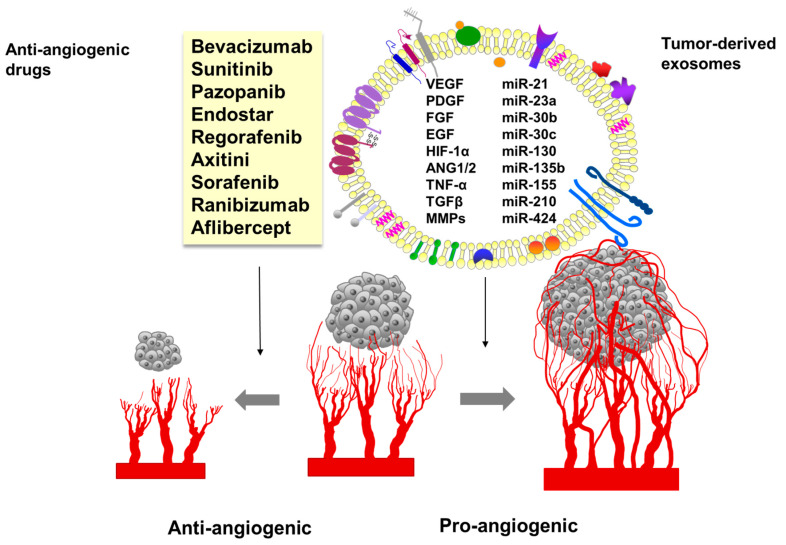
Exosomes as carriers of pro-angiogenic factors and anti-angiogenic drugs.

In remodeling of the tumor microenvironment, inducing angiogenesis, numerous proteins transported by TEX are involved (Figure 4). The molecular and genetic cargo of TEX is responsible for phenotypic and functional reprogramming of endothelial cells and other normal cells residing in the TME [98,156]. It was shown that TEX-driven cross-talk of MSCs with immune cells blocks their anti-tumor activity and/or converts them into suppressor cells [157].

In exosomes derived from glioblastoma cells, seven proangiogenic proteins were found: angiogenin, VEGF, fibroblast growth factor (FGF), interleukin-6, interleukin-8 and tissue inhibitors of metalloproteinases 1 and 2 (TIMP-1 and TIMP-2). These proteins are involved in angiogenesis and increased malignancy in this tumor [154]. The presence of pro-angiogenic VEGF and IL-6 has also been found in melanoma-derived exosomes [158]. In turn, CD147-positive exosomes derived from epithelial ovarian cancer cells can promote the angiogenic phenotype in endothelial cells in vitro [159]. Lang et al. showed that gliomas can induce angiogenesis by secreting linc-POU3F3-rich exosomes [160]. Angiogenesis is also promoted by TEX enriched in matrix metalloproteinases, especially MMP-2, MMP-9 and MMP-13. These proteins have been found in glioblastoma-, melanoma-, myeloma- and nasopharyngeal carcinoma-derived exosomes [154,158,161,162,163,164]. Exosomes isolated from the peritoneal fluid of patients with colorectal cancer and from pancreatic adenocarcinoma cell cultures contains significant amounts of tetraspanin 8 (Tspan8), which promotes cancer metastasis and angiogenesis [165,166]. Another proangiogenic protein is annexin II (Anx II), whose presence has been demonstrated in exosomes derived from breast cancer cells. This protein, acting as a co-receptor for tissue plasminogen activator (tPA), plays an important role in tumor neoangiogenesis [167]. TEX are also RNA carriers. Lang et al. showed that intergenic non-coding RNA POU3F3 (linc-POU3F3) present in glioma cell-derived exosomes is involved in the process of neoangiogenesis [168].

The angiogenic potential of TEX depends on the conditions under which they are secreted. Studies on the effects of esophageal squamous cell carcinoma (ESCC) exosomes on the ability to form vessels by human umbilical vein endothelial cells (HUVECs) showed that HUVEC cultured with exosomes secreted under low oxygen conditions showed a better ability to form vessels compared to those grown from normal exosomes [169]. Exosomes secreted by cancer cells under hypoxic conditions are enriched in micro-RNAs, such as miR-135b, miR-210, miR-21, miR-30b, miR-30c, and miR-424. These exosomes promote angiogenesis through suppressing expression of factor-inhibiting HIF-1, promoting hypoxic signaling, or upregulating the expression of proangiogenic factors [170]. Exosomal miR-23a derived from hypoxic lung cancer cells also stimulates angiogenesis by targeting prolyl hydroxylase and the tight junction protein ZO-1 [171]. The involvement of miR-23a in angiogenesis has also been demonstrated in hypoxic HCC [172]. It has been shown that exosomes containing miR-155 secreted by gastric cancer (GC) cells significantly increase the rate of tumor angiogenesis by enhancing the expression of VEGF [173]. Skin cancer-derived exosomes and melanoma-derived exosomes can promote angiogenesis by delivering miR-9 to endothelial cells and activating the JAK–STAT pathway [174,175]. Research by Yang et al. showed that exosomes secreted by miR-130-rich gastric cancer cells inhibited c-myb gene expression in vascular endothelial cells, promoting angiogenesis and tumor growth [176]. In turn, research by Zhang et al. showed that exosomes transporting hepatocyte growth factor siRNA (HGF siRNA) exerted an inhibitory effect on angiogenesis and tumor growth in gastric cancer [177].

## 10. The Role of Exosomes in Resistance to Anti-Angiogenic Therapies

Despite the fact that anti-angiogenesis therapies may prolong progression-free survival (PFS), they have a limited impact on overall survival (OS) and do not constitute a permanent cure in renal cell carcinoma (RCC), colorectal cancer (CRC), or breast cancer (BC) [178,179,180,181]. This lack of clinical benefit can be put down to preexisting resistance or rapid adaptation to anti-angiogenic agents. Multiple resistance mechanisms against AIs exist. They include the upregulation of alternative angiogenic factors by tumor cells, the involvement of stromal cells, and co-option/mimicry. Sunitinib, a multi-targeted receptor tyrosine kinase inhibitor, is one of the first line agents for patients with advanced RCC. However, intrinsic or acquired resistance to sunitinib has become a major issue for treatment [182]. LncARSR was identified as a mediator of sunitinib resistance by Qu et al. in renal cell carcinoma. By acting as a competing endogenous RNA for miR-34 and miR449, it increases the expression of AXL and c-MET targets, showing that the exosome-mediated transmission of lncARSR can confer resistance to sensitive cells [183]. They also found that lncARSR could be secreted from resistant cells via exosomes, transforming sunitinib-sensitive cells into resistant cells, thereby disseminating drug resistance [183]. RAB27B is a leading protein involved in exosome secretion [184]. Oncogenic effects have been reported in several cancers [185,186,187]. RNA sequence and pathway analysis has suggested that the oncogenic effects of RAB27B could potentially be associated with mitogen-activated protein kinase (MAPK) and VEGF signaling pathways [188]. These results have shown that RAB27B is a prognostic marker and a novel therapeutic target in sunitinib-sensitive and -resistant RCCs [188]. Placental growth factor (PlGF) is a member of the VEGF subfamily that binds to VEGFR-1 and its co-receptors, NRP-1 and 2. PlGF/VEGFR-1 signaling activates the downstream PI3K/Akt and p38 MAPK pathways independent of VEGF-A signaling [189,190]. Anaplastic lymphoma kinase (ALK), in exosomes secreted by BRAF inhibitor-resistant melanoma cells, has recently been demonstrated to transfer drug resistance by activating the MAPK signaling pathway in recipient cells [191]. Furthermore, in pancreatic cancer, EVs released by upregulated RAB27B have been shown to activate p38 MAPK [192]. Alterations in EVs produced by glioblastoma cells following bevacizumab treatment were described by Simon and colleagues [193]. Interestingly, bevacizumab, which is able to neutralize glioblastoma cell-derived VEGF-A, was found to be directly captured by glioblastoma cells and sorted at the surface of respective EVs. It was observed that treatment with bevacizumab induces changes in the proteomic content of EVs, which is associated with tumor progression and therapeutic resistance. Accordingly, the glioblastoma cells inhibiting EV production improved the anti-tumor effect of bevacizumab. Together, this data underlines the potential new mechanism of glioblastoma escape from bevacizumab activity [193]. Moreover, cetuximab, an EGF-R monoclonal IgG1 antibody, has been observed to be associated with EVs derived from treated cancer cells, suggesting that such processes could be implicated in the limited response of tumors to therapy [194]. 

## 11. Exosomes as Drug Carriers of Anti-Cancer Therapy 

Exosomes have recently turned out to be possible natural carriers of therapeutic agents for cancer therapy [195]. Exosomes are formed by a lipid bilayer delimiting an aqueous core. This allows delivery of hydrophobic and hydrophilic drugs, thus increasing versatility [196]. Although many body cells produce exosomes, mesenchymal stem cells (MSCs) are among the most prolific. Therefore, they are more suited to mass production of exosomes for drug delivery [197]. Clinical trials of MSC-derived exosomes that are currently underway focus on gene delivery, regenerative medicine, and immunomodulation [198]. It is believed that MSC-derived exosomes have intrinsic homing capabilities, similar to those of MSCs. In cancer treatment, they can penetrate the tumor site [199]. Similarly, MSC-derived exosomes could potentially target hypoxia, as hypoxia is a potent mediator that directs exosome migration [200]. It has been observed in hypoxia studies that hypoxic cancer cells avidly uptake exosomes that are produced in hypoxic conditions [201]. It has been shown that miRNA-enclosed exosomes derived from cancer cells could interact with endothelial cells and thereby stimulate endothelial cell proliferation, migration and tube formation [202]. Some studies suggest that miRNA may be valuable as novel targets for the treatment of carcinoma. A Chinese group identified that exosomal miR-9 derived from nasopharyngeal carcinoma cells inhibits angiogenesis by targeting Midkine (a heparin-binding growth factor) and regulating the PDK/AKT pathway in nasopharyngeal carcinoma [203]. The findings of this study suggest that miR-9 and Midkine may be valuable as novel targets for the treatment of human nasopharyngeal carcinoma [203]. Circulating microRNAs (miRNAs) in exosomes are used as functional biomarkers for diagnostics and prognostics, while synthetic miRNAs in exosomes could be applicable for therapeutics.

As increased PD-L1 expression was observed after anti-angiogenic treatment, Allen et al. treated refractory pancreatic, breast and brain tumor mouse models with combined therapy using PD-1/PD-L1 pathway blockers and anti-angiogenic agents to increase the efficacy of anti-angiogenic therapy based on VEGF and VEGFR-2 inhibition. They showed that anti-PD-1 therapy sensitized and prolonged the efficacy of the anti-angiogenic therapy in pancreatic and breast cancer models. Moreover, the anti-angiogenic therapy improved anti-PD-L1 treatment, especially by increased cytotoxic T cell infiltration [204].

It was shown that chemotherapy stimulates secretion of exosomes and alters exosome composition. Exosomes secreted during therapy can be transferred to both tumor and host cells, altering their behavior and enhancing tumor survival and progression [205]. On the other hand, cancer cell-derived EVs may be used as effective carriers of drugs such as paclitaxel, increasing their cytotoxicity [206].

Enhanced permeability and retention (EPR) effect-based nanomedicine, based on tumor blood flow, is a promising strategy for successful anticancer therapy [207,208]. Tumor blood flow is frequently obstructed as tumor size increases because advanced large tumors show heterogeneity in the EPR effect. Accordingly, it would be very important to apply enhancers of the EPR effect in the clinical setting to make the EPR effect more uniform [209,210].

## 12. Conclusions and Outlook

Angiogenesis is controlled by various angiogenic and anti-angiogenic factors, which are carried by exosomes. The imbalance between these factors leads to dysregulation of angiogenesis during development of tumors. Effective inhibition of tumor angiogenesis might arrest tumor progression. Combined strategies based on angiogenesis inhibitors and immunotherapy effectively enhances the benefits of therapies in cancers [211]. Angiogenesis-targeted therapy of cancer is considered a promising strategy for management of cancer progression, but effective treatments requires further research. An increased understanding of the cross-talk between tumor cells, endothelial cells, and immune cells during immune checkpoint blockade therapy may lead to new combinatorial treatment regimens [212]. Control of exosome composition may serve as an effective strategy to augment the long-term efficacy of anti-angiogenic therapies for tumors. Given the molecular complexity of angiogenesis, a better understanding of how exosomes participate in this process represents an important challenge, which can open new paths for the development of novel and effective anti-angiogenic drugs. To clarify the molecular mechanisms by which exosomal miRNAs, mRNAs, and proteins inhibit angiogenesis in endothelial cells and transmit drug-resistance, in silico analysis should be performed to predict possible miRNA, mRNA, and protein targets using database resources. Most importantly, the clinical relevance of exosomal molecules in cancer patients awaits further validation in larger samples sizes.

## Figures and Tables

**Table 1:** Review of randomized phase III trials examining the effect of anti-angiogenic therapy in different types of cancers.

Type of Cancer	Angiogenesis Inhibitor Used	Targeting Factor/Regulator	Combination with other Drugs/Alone	Number of Patients	Patient Outcome [Reference]
Prostate cancer	Bevacizumab	VEGF-A	Docetaxel, Prednisone	1050	No improvement in overall survival [117]
Prostate cancer	Aflibercept	circulating VEGF-A	Docetaxel, Prednisone	1224	No improvement in overall survival [135]
Prostate cancer	Sunitinib	Receptor tyrosine kinase	Prednisone	873	No improvement in overall survival [136]
Prostate cancer	Lenalidomide	Multiple, (e.g., VEGF-induced PI3K-Akt pathway signalling)	Docetaxel, Prednisone	1059	Worse overall survival [137]
Hepatocellular carcinoma	Sorafenib	VEGF-2, PDGFR-β, and other signaling cascades	Alone	602	3 months longer median survival [138]
Hepatocellular carcinoma	Levatinib	VEGFR, FGF receptor, PDGF receptor	Alone	1492	Median survival time non-inferior to sorafenib [139]
Hepatocellular carcinoma	Regorafenib	Tyrosine kinase	Alone	573	Survival benefit in HCC patients progressing on sorafenib [121]
Hepatocellular carcinoma	Cabozantinib	VEGFR 1, 2, and 3, MET, and AXL	Alone	707	Survival benefit in HCC patients progressing on sorafenib [122]
Hepatocellular carcinoma	Ramucirumab	VEGFR-2	Alone	292	Survival benefit in HCC patients progressing on sorafenib [140]
Melanoma	Sorafenib	VEGF-2, PDGFR-β, and other signalling cascades	Carboplatin, Paclitaxel	823	No improvement in overall survival [141]
Ovarian cancer	Bevacizumab	VEGF-A	Paclitaxel, Carboplatin	674	Improvement in overall survival [142]
Colorectal cancer	Bevacizumab	VEGF-A	FOLFOX-4, XELOX	820	Improvement in overall survival [143]
Colorectal cancer	Aflibercept	VEGF-A, VEGF-B, and PlGF	FOLFIRI	1226	Improvement in overall survival [144]
Colorectal cancer	Ramucirumab	VEGF receptor 2	FOLFIRI	1072	Improvement in overall survival [145]
Breast cancer	Bevacizumab	VEGF-A	Paclitaxel	673	No improvement in overall survival [146]
Lung cancer	Bevacizumab	VEGF-A	Cisplatin, Pemetrexed	376	Improvement in overall survival [147]
Lung cancer	Atezolizumab, Bevacizumab	PD-L1, VEGF-A	Carboplatin, Paclitaxel	1202	Improvement in overall survival [148]
Pancreatic cancer	Bevacizumab	VEGF-A	Gemcitabine, Erlotinib	607	No improvement in overall survival [149]
Glioblastoma	Bevacizumab	VEGF-A	Lomustine	437	No improvement in overall survival [150]

Abbreviations: VEGF-A = vascular endothelial growth factor A; PI3K-Akt = Phosphatidylinositol-3-kinase and protein kinase B; PDGFR-β = platelet-derived growth factor receptor β; FGF = fibroblast growth factor; AXL = derived from the Greek word “anexelekto”, meaning uncontrolled, also known as Ark, Tyro7 and Ufo; FOLFOX = fluorouracil, folinic acid and oxaliplatin; XELOX = capecitabine plus oxaliplatin; PlGF = placental-derived growth factor; FOLFIRI = fluorouracil, folinic acid and irinotecan; PD-L1 = programmed death ligand-1.
